# mHealth Intervention is Effective in Creating Smoke-Free Homes for Newborns: A Randomized Controlled Trial Study in China

**DOI:** 10.1038/s41598-017-08922-x

**Published:** 2017-08-31

**Authors:** Shaohua Yu, Zongshuan Duan, Pamela B. Redmon, Michael P. Eriksen, Jeffrey P. Koplan, Cheng Huang

**Affiliations:** 1 Department of Criminal Justice and Criminology, Georgia State University, Atlanta, GA 30303 USA; 2 School of Public Health, Georgia State University, Atlanta, GA 30303 USA; 3 Global Health Institute, Emory University, Atlanta, GA 30322 USA; 4 Department of Global Health, George Washington University, Washington, DC 20052 USA; 5 School of Economics and Management, Harbin Institute of Technology (Shenzhen), Shenzhen, Guangdong 518055 China

## Abstract

Mobile-phone-based smoking cessation intervention has been shown to increase quitting among smokers. However, such intervention has not yet been applied to secondhand smoke (SHS) reduction programs that target smoking parents of newborns. This randomized controlled trial, undertaken in Changchun, China, assessed whether interventions that incorporate traditional and mobile-phone-based education will help create smoke-free homes for infants and increase quitting among fathers. The results showed that the abstinence rates of the fathers at 6 months (adjusted OR: 3.60, 95% CI: 1.41–9.25; p = 0.008) and 12 months (adjusted OR: 2.93, 95% CI: 1.24–6.94; p = 0.014) were both significantly increased in the intervention group compared to the control. Mothers of the newborns in the intervention group also reported reduced exposure to SHS at 12 months (adjusted OR: 0.53, 95% CI: 0.29–0.99; p = 0.046). The findings suggest that adding mHealth interventions to traditional face-to-face health counseling may be an effective way to increase male smoking cessation and reduce mother and newborn SHS exposure in the home.

## Introduction

Second-hand smoke (SHS) exposure is a major, yet preventable, threat to infant and child health. It puts newborns at risk of developing sudden infant death syndrome, and increases susceptibility to lower respiratory infections, middle ear infections, childhood cancers, asthma, allergies, reduced physical development, and decreases in cognition and behavior for infants and children^1–7^. Evidence suggests that exposure to SHS, mostly occurring in the home, threatens the health of roughly 40% of children worldwide^8^.

In China, most women do not smoke (<3%)^9^ yet a large number of infants are still exposed to SHS in the home due to the high adult male smoking rate (>50%)^10^. Estimates of the prevalence of household SHS exposure among non-smoking pregnant women in China range from 58% to 73%^11^; levels of exposure remain high after babies are born^12^. These high exposure rates among pregnant women underscore the fact that at least half of all newborns in Chinese families are regularly exposed to SHS in the home. The risks associated with exposure to SHS among infants and the high exposure rates in the home due to the father’s smoking status creates an urgent need to create smoke-free homes for the health of infants in China.

For Chinese families with infants and children to maintain a smoke-free home, fathers would have to quit smoking or smoke only outside of the home. However, the quit rate among men in China is low^13^. According to the Global Adult Tobacco Survey (GATS), Chinese smokers were among the most resistant to quitting in the world, with more than 80% reporting having no intention to quit smoking^14^. Chinese women have traditionally accepted the social norm of men smoking at their presence^15^, and have had limited success with stopping in-home smoking due to the desire to maintain household harmony^16^. Studies have found that only a small portion of families in China have strict home smoking restrictions (<15%). For households with regular smokers, usually husbands and/or fathers, the rate of complete smoking bans is as low as 3%^17^. Without interventions targeted towards reducing in-home smoking, children and infants will remain exposed to high levels of SHS.

Previous smoking cessation and SHS reduction intervention programs that targeted smoking parents have relied heavily on face-to-face counseling and distribution of self-help materials^18–22^. In these studies, standard health advice was usually provided by health professionals and supplemented with printed health education materials; one study utilized follow-up telephone support^19^. A recent meta-analysis found that traditional interventions were not effective in reducing smoking rate among parents of children under the age of 4^21^. Many other shortcomings of traditional methods are apparent as well. For instance, the amount of educational information, motivational messages, and behavior-change guidance that can be conveyed during physician consultations is limited^23^. The time when and the location where the participants can be reached by health professionals are restricted too, and hence, it is costly to maintain routine communication with the participants. New technology is clearly needed for improved delivery of existing health services.

The rapid increase in the number of people owning a mobile phone has led to the incorporation of mobile health (mHealth) interventions into traditional health practices^23^. Mobile phones are a flexible, accessible, and low cost method for delivering health promotion interventions. mHealth interventions allow for the conveyance of information, triggers, and support whenever clients carry a mobile phone, and embrace the element of anonymity^24^. In addition, these interventions can be scaled to large populations and personalized to meet individual needs^25^. The application of mHealth interventions, in particular short message service (SMS), has shown improved outcomes in smoking cessation interventions^26–28^. According to a recent review of 13 randomized controlled trials, smoking quit rates were significantly higher for the text messaging intervention group than the control group [Odds Ratio (OR): 1.35, 95% Confidence Interval (CI): 1.23–1.48]^26^.

To our knowledge, mobile-phone-based health interventions have not yet been applied to household SHS reduction programs that target smoking parents of young children. We also found an absence of any experimental research studies aimed at creating smoke-free homes for infants in China. To fill these knowledge gaps, we designed a randomized controlled trial (RCT) that aimed to assess whether or not interventions incorporating traditional and mobile-phone-based education help create smoke-free homes for infants and increase quitting among fathers in Chinese households. In addition, our study included non-smoking spouses (wives/mothers) based on the hypothesis that spousal pressure and support could better help convince male smokers to quit for the health of their children.

## Methods

### Study design and participants

The Smoke-free Homes mHealth Intervention Project was a single-blind, randomized controlled trial of an SHS reduction and smoking cessation service enhanced with mobile phone text messages. The intervention was implemented in Changchun, China and managed by Changchun Health Education Institute and the China Tobacco Control Partnership^29^. The recruitment of participants was completed over a one-month period (August 25 to September 28, 2014). The protocol and survey questionnaires of the study were approved by Jilin University Second Hospital Institutional Review Board (IRB). All methods included in the study were consistent with the principles of the Declaration of Helsinki.

Families were eligible for inclusion if they met the following criteria: nonsmoking mothers and their newborns were currently exposed to SHS in the home; fathers currently smoked cigarettes in the home; the parents both owned a mobile phone that could receive text messages; and the family was able to provide informed consent. There were no restrictions for trial participants on the use of other smoking cessation services or additional support.

The screening process took place in 15 local maternal-child health centers that were selected as cooperating organizations of our intervention project. Trained health workers in those centers asked all mothers attending their initial post-delivery visit (1 month after birth) to complete a short health questionnaire with questions related to tobacco use and household SHS exposure. Among all eligible families, 342 were recruited as participants for the trial.

### Interventions

Participant families were randomly allocated to one of the following three groups: Intervention Group A (I-A), Intervention Group B (I-B), or the Control Group. Randomization was fully computerized, using no blocks or strata, and each participant was allocated a number 1 (then assigned to I-A), 2 (then assigned to I-B), or 3 (then assigned to control group) with equal probability.

Three home visits were conducted with all participants. The initial visit, conducted in December 2014, assessed baseline SHS levels in the home. The parents were asked to complete short surveys on smoking, exposure to SHS, and knowledge of the harms of smoking and SHS. The husband was asked additional questions about his smoking habits and quit history. At this visit, participants allocated to I-A received in-person counseling from the trained health care workers on the harms of SHS to infants; education on establishing a smoke-free home, including a manual with step-by-step instructions; and table tents and posters to display in the home to encourage fathers and other visitors not to smoke. The smoke-free homes manual provided a 5-step plan for creating a smoke-free home with information on: (1) deciding to create a smoke-free home; (2) talking to family members; (3) setting a date for going smoke-free; (4) actually creating a smoke-free home; and (5) keeping the home smoke-free. Participants allocated to I-B received the same educational intervention and materials as I-A at this visit, and also received a text message intervention in the coming months. The text message intervention included messages to the mother and her husband on the harms of SHS to the mother and the infant. The husband received additional cessation text messages to encourage him to quit smoking. Text message examples are listed in Table 1. A total of 9,500 messages were sent to participants in I-B between January 10^th^ and February 22^nd^ in 2015. The control group received only standard care for their initial postnatal visits, which did not include any tobacco control and cessation counseling service.

**Table 1:** Examples of mHealth intervention text messages.

**Type**	**Messages**
Health effects of smoking and quitting	Smoking damages your lungs, increases the risk of lung infection, and causes lung disease.
	According to the China Report on Health Hazards of Smoking, the average life expectancy of smokers is 10 years shorter than that of non-smokers
	Cigarettes affect your teeth, gums, and breath. People will start to notice your new bright smile.
	Did you know smoking can also make your bones weak and easier to break?
	Quitting smoking reduces the chances of getting many diseases, such as lung cancer, heart disease, and lung disease. And quitting improves the chance of successful treatment if you already have a disease.
	Your lung capacity increases by as much as 30% after a few weeks without cigarettes. Your body starts to heal within days of quitting smoking.
Health effects of exposure to SHS	Your family can get sick from your smoking. Think again before you smoke.
	Exposure to tobacco smoke seriously endangers children’s health and can causes severe ear infections, asthma, poor lung function, and, and sudden infant death
	Breathing tobacco smoke is harmful to health. Even occasional breathing of tobacco smoke can cause serious health problems.
	Setting up exhaust fans and other ventilation devices indoors cannot prevent the harm of smoke exposure. The only way that non-smokers can avoid the hazards from smoke exposure is to make their environment completely smoke-free.
	Smoking in cars is dangerous. Even with the windows rolled down, you and your passengers will still inhale dangerous second hand smoke.
	Breathing other people’s cigarette smoke causes many diseases, including lung cancer, breast cancer, and heart diseases.

Follow-up home visits were performed at 6 and 12 months. Follow-up self-reported data on SHS levels and smoking behavior were collected on-site. Participants in I-A and I-B received additional counseling at the six-month follow-up visit if they had been unable to successfully create a smoke-free home since the initial visit.

Newborn’s parents were required to complete an onsite questionnaire survey at baseline, 6 and 12 months. The questionnaire was initially developed in English, and translated into Chinese to onsite survey. Both questionnaires were tested among Emory University students who were fluent in both languages. The questionnaire included questions on demographic information, smoking status, exposure to SHS at home, and perceived health risks of smoking and SHS exposure. Major outcomes included fathers’ abstinence rate and mothers’ SHS exposure at home at baseline, 6 months and 12 months. Fathers’ self-reported smoking status at home and mothers’ exposure to SHS at home in the last 6 month were compared and matched to determine fathers’ current smoking status and infants SHS exposure at home. If mothers reported exposing to SHS due to fathers smoking at home in the last 6 month, the father will be classified as smoking regardless of his self-reported status.

### Sample size and statistical analysis

Based on 80% power and 5% level of significance, we calculated that 300 participants (100 in each group) were needed to detect a moderate to large effect size at the end of the program, given an expect dropout rate of 20%.

The primary outcomes of the study were self-reported smoking status among the fathers and self-reported SHS exposure at home among the mothers at the 6- and 12-month follow-up periods. Other outcomes included: fathers’ self-reported intent to quit, knowledge of SHS and tobacco smoking, and smoke-free home policy enforcement at the 6- and 12-month follow-up periods. For each outcome, a multivariate logistic regression was performed to determine how each intervention affected the outcome variable, controlling for demographic and socio-economic variables including age, ethnicity, education level and occupation. Data were analyzed using SAS 9.3 (SAS Institute, Cary, NC). Estimation of relative risks, 95% confidence intervals, and two-sided p values at 6 and 12 months were reported.

### Ethical approval and informed consent

The protocol of the study was approved by the Changchun Institutional Review Board (IRB).The written consent forms were distributed to all participants. The study participants provided written consent to participate.

### Clinical Trial Registration

This study has been registered as a randomized clinical trial (RCT) at the Chinese Clinical Trial Registry (ChiCTR) with registration number as ChiCTR-OIC-17010803 on March 6, 2017.

## Results

**Figure 1:**
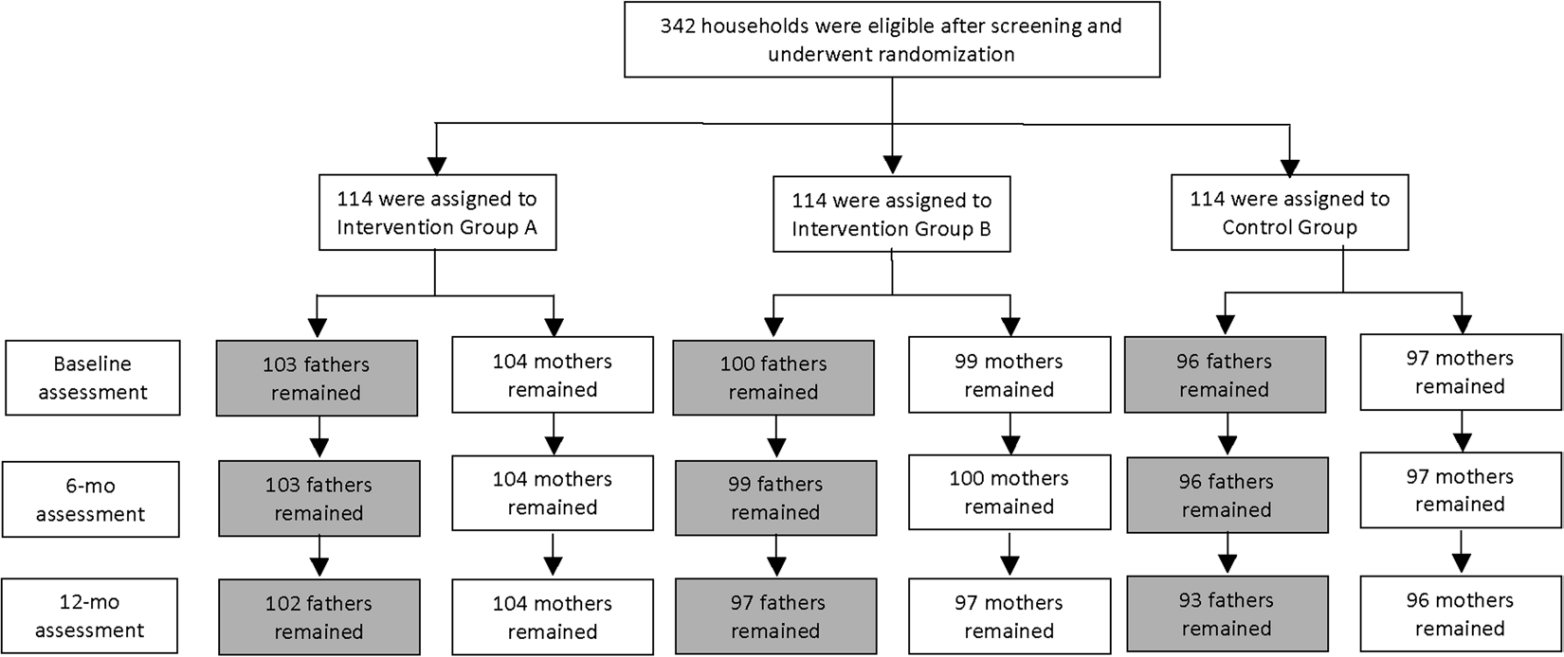
Flowchart of participant selection process.

The first round of recruitment included 342 mothers who were randomly assigned to I-A, I-B, or the control group (Fig. 1). Three hundred and four completed the baseline survey, with 106 (93%), 100 (88%), and 98 (86%) in I-A, I-B, and the control group, respectively. Three participants dropped out of the trial at the 6-month follow-up, two from I-A and one from the control group. Another four participants were lost at the 12-month follow-up, including three from I-B and one from the control group.

Fathers of the newborns (n = 342) were also assigned to the three groups, corresponding to the assignment of their wife; 103 (90%), 100 (88%), and 96 (84%) were allocated to I-A, I-B, and the control group, respectively, to complete the baseline surveys. At the 6-month follow-up, 298 fathers completed the surveys. The I-B group lost one participant. At 12 months, 292 fathers completed the follow-up surveys (participants lost to follow-up: one in I-A, two in I-B, and three in the control group).

We conducted a t-test and found no significant differences in the characteristics between those who lost to follow up and those who remained in the sample (the results are available upon request), and we therefore handle the missing data with listwise deletion in the analysis.

### Baseline characteristics

All baseline characteristics are shown in Table 2. At baseline, the average age of the recruited fathers was 31.8 (SD: 4.5). All fathers had received some education, with 69% having completed community college education or above. More than ninety-six percent were currently employed. All participant fathers were smokers; 232 (76.6%) smoked daily, and 92 (30.8%) smoked more than 15 cigarettes per day. When asked about their quit history, 113 (37.8%) participant fathers stated that they had tried to quit smoking in the past 12 months. There were no significant differences between groups for any socio-demographic characteristics or smoking variables.

**Table 2:** Participants baseline characteristics.

	Fathers					Mothers				
	Total (%) N = 299	I-A (%) n = 103	I-B (%) n = 100	Control (%) n = 96	P	Total (%) N = 300	I-A (%) n = 104	I-B (%) n = 99	Control (%) n = 97	P
***Sociodemographic***										
Mean Age (STD)	31.8 (4.5)	31.8 (4.8)	31.9 (4.1)	31.6 (4.5)	0.962	29.6 (3.8)	29.6 (3.8)	29.9 (3.8)	29.3 (3.7)	0.985
Ethnicity										
Han	290 (97.0)	100 (97.1)	97 (97.0)	93 (96.9)	0.996	287 (95.7)	99 (95.3)	94 (95.0)	94 (96.9)	0.767
Other	9 (3.0)	3 (2.9)	3 (3.0)	3 (3.1)		13 (4.28)	5 (4.72)	5 (5.0)	3 (3.1)	
Education										
Illiterate	0 (0)	0 (0)	0 (0)	0 (0)	0.727	0 (0)	0 (0)	0 (0)	0 (0)	0.491
High school or lower	93 (31.1)	38 (36.9)	29 (29.0)	26 (27.1)		92 (30.7)	36 (34.6)	27 (27.3)	29 (29.9)	
Community college	111 (37.1)	35 (34.1)	36 (36.0)	40 (41.7)		119 (39.7)	42 (40.4)	41 (41.4)	36 (37.1)	
Bachelor degree	88 (29.4)	28 (27.2)	33 (33.0)	27 (28.1)		79 (26.3)	24 (23.1)	29 (29.3)	26 (26.8)	
Graduate degree	7 (2.3)	2 (1.9)	2 (2.0)	3 (3.1)		10 (3.3)	2 (1.9)	2 (2.0)	6 (6.2)	
Current Occupation										
Peasant/fisherman	0 (0)	0 (0)	0 (0)	0 (0)	0.450	2 (0.7)	0 (0)	0 (0)	2 (2.1)	0.499
Worker/service personnel	123 (41.1)	40 (38.8)	42 (42.0)	41 (42.7)		73 (24.3)	23 (22.1)	25 (25.3)	25 (25.8)	
Cadre/specialist	19 (6.4)	10 (9.7)	6 (6.0)	3 (3.1)		21 (7.0)	9 (8.7)	9 (9.1)	3 (3.1)	
Privately employed	108 (36.1)	36 (35.0)	35 (35.0)	37 (38.5)		57 (19.0)	19 (18.3)	17 (17.2)	21 (21.7)	
Retired	0 (0)	0 (0)	0 (0)	0 (0)		0 (0)	0 (0)	0 (0)	0 (0)	
Unemployed	11 (3.7)	3 (2.9)	2 (2.0)	6 (6.3)		76 (25.3)	25 (24.0)	26 (26.3)	25 (25.8)	
Student/householder	0 (0)	0 (0)	0 (0)	0 (0)		42 (14.0)	14 (13.5)	13 (13.1)	15 (15.5)	
Other	38 (12.7)	14 (13.6)	15 (15.0)	9 (9.4)		29 (9.7)	14 (13.5)	9 (9.1)	6 (6.2)	
***Smoking and SHS exposure***										
Smoking Status										
Daily	232 (76.6)	83 (80.6)	79 (79.0)	70 (72.9)	0.396					
Less than daily	67 (22.1)	20 (19.4)	21 (21.0)	26 (27.1)						
Not at all	0 (0)	0 (0)	0 (0)	0 (0)						
Number of cigarettes smoked per day										
Light smoking (<=15)	207 (69.2)	76 (73.8)	63 (63.0)	68 (70.8)	0.244					
Moderate smoking (16-24)	80 (26.8)	26 (25.2)	29 (29.0)	25 (26.0)						
Heavy smoking (>=25)	12 (4.0)	1 (1.0)	8 (8.0)	3 (3.1)						
Ever tried to quit	163 (54.5)	61 (59.2)	52 (52.0)	50 (52.1)	0.495					
Tried to quit in last 12 months	113 (37.8)	42 (40.8)	35 (35.0)	36 (37.5)	0.696					
Exposure to household SHS last week										
Almost everyday						185 (61.7)	65 (62.5)	66 (66.7)	56 (57.7)	0.278
Over three days per week						36 (12.0)	13 (12.5)	7 (7.1)	14 (14.4)	
1–3 days per week						50 (16.7)	16 (15.4)	13 (13.1)	20 (20.6)	
None						29 (9.7)	10 (9.6)	12 (12.1)	7 (7.2)	

The average age of participant mothers was 29.6 (SD: 3.8). Almost seventy percent had completed community college education or above, and 25.3% were unemployed. When asked about household SHS exposure, 185 (61.7%) participants reported exposed to SHS in the home almost every day. No significant differences existed between groups for any baseline characteristics.

### Outcome analyses

The primary outcomes assessed were self-reported smoking cessation among fathers and self-reported exposure to household SHS among mothers of the newborns at 6 and 12 months post-randomization. Results showed that the crude odds ratios are similar to adjusted odds ratios in all tables, which is typical in a randomized controlled trial study. We hereafter report adjusted odds ratios only. Table 3 showed that participant father self-reported smoking abstinence at 6 months was significantly increased in I-B compared to the control group (20.0% vs.7.3% control; adjusted odds ratio (OR):3.60, 95% CI: 1.41–9.25; p = 0.008). Smoking abstinence at 12 months was 22.7% in I-B compared to9.7% in the control group (adjusted OR: 2.93, 95% CI: 1.24–6.94; p = 0.014) (Table 4). No significant difference was detected between I-A and the control group or between I-B and I-A. Although no reduction of the self-reported exposure rate to SHS among mothers of newborns was found at 6 months (Table 5), the rate at 12 months was significantly decreased in I-B compared to the control group (60.8% vs. 75.0% control; adjusted OR: 0.53, 95% CI: 0.29–0.99; p = 0.046) (Table 6).

**Table 3:** Intervention effects on smoking cessation, smoke-free homes enforcement, and other related outcomes among fathers of newborn at 6 months.

	N (%)				Crude OR (95% CI), p			Adjusted OR^ (95% CI), p		
	I-A (n = 103)	I-B (n = 99)	Control (n = 96)	I-B vs I-A		I-B vs Control	I-A vs Control	I-B vs I-A	I-B vs Control	I-A vs Control
***Primary Outcomes***										
Self-reported quitting	11 (10.7)	20 (20.0)	7 (7.3)	2.12		3.22	1.52	2.21	3.60	1.63
				(0.96, 4.69), 0.064		(1.29, 8.02), 0.012*	(0.56, 4.1), 0.408	(0.97, 5), 0.059	(1.41, 9.25), 0.008**	(0.59, 4.53), 0.345
***Other Outcomes***										
Smoking never permitted inside home	21 (20.4)	26 (26.3)	16 (16.7)	1.34		1.68	1.25	1.36	1.76	1.3
				(0.7, 2.57), 0.372		(0.84, 3.34), 0.142	(0.62, 2.52), 0.538	(0.71, 2.61), 0.358	(0.88, 3.54), 0.109	(0.64, 2.64), 0.470
You or others smoke at home	61 (59.2)	59 (60.0)	55 (57.3)	1.06		1.13	1.06	1.09	1.13	1.05
				(0.61, 1.86), 0.828		(0.64, 1.99), 0.681	(0.61, 1.85), 0.841	(0.62, 1.91), 0.777	(0.64, 2.01), 0.667	(0.60, 1.84), 0.877
I don’t want to maintain smoke-free home (SFH)	26 (25.2)	22 (22.2)	23 (24.0)	0.6		0.75	1.25	0.62	0.76	1.26
				(0.35, 1.02), 0.061		(0.43, 1.29), 0.302	(0.74, 2.10), 0.410	(0.36, 1.03), 0.065	(0.45, 1.30), 0.320	(0.77, 2.14), 0.430
SHS causes heart disease in adults	87 (84.5)	84 (84.9)	77 (80.2)	1.01		1.36	1.36	1.00	1.35	1.35
				(0.47, 2.16), 0.986		(0.65, 2.87), 0.413	(0.65, 2.82), 0.415	(0.47, 2.17), 0.993	(0.64, 2.86), 0.428	(0.64, 2.82), 0.427
SHS causes lung illnesses in children	101 (98.1)	97 (98.0)	92 (95.8)	0.96		2.11	2.20	0.89	2.09	2.36
				(0.13, 6.95), 0.968		(0.38, 11.79), 0.396	(0.39, 12.27), 0.370	(0.12, 6.50), 0.904	(0.37, 11.83), 0.404	(0.42, 13.37), 0.331
Smoking causes stroke	70 (68.0)	71 (71.7)	66 (68.8)	1.20		1.15 (0.62,	0.96	1.12	1.12	1.01
				(0.65, 2.18), 0.561		(0.53, 2.13), 0.651	(0.60, 1.75), 0.905	(0.60, 2.06), 0.723	2.10), 0.714	(0.55, 1.85), 0.985
Smoking causes heart attack	83 (80.6)	83 (83.8)	77 (80.2)	1.25		1.28	1.02	1.17	1.25	1.07
				(0.61, 2.58), 0.546		(0.61, 2.67), 0.510	(0.51, 2.06), 0.947	(0.56, 2.44), 0.674	(0.59, 2.62), 0.561	(0.52, 2.17), 0.862

**p < 0.01. *p < 0.05.

^Controlling age, ethnicity, education level and occupation.

**Table 4:** Intervention effects on smoking cessation, smoke-free homes enforcement, and other related outcomes among fathers of newborn at 12 months.

	N (%)			Crude OR (95% CI), p			Adjusted OR^ (95% CI), p		
	I-A (n = 102)	I-B (n = 97)	Control (n = 93)	I-B vs I-A	I-B vs Control	I-A vs Control	I-B vs I-A	I-B vs Control	I-A vs Control
***Primary Outcomes***									
Self-reported quitting	17 (16.7)	22 (22.7)	9 (9.7)	1.47	2.74	1.87	1.38	2.93	2.13
				(0.72, 2.97), 0.287	(1.19, 6.31), 0.018*	(0.79, 4.42), 0.156	(0.67, 2.84), 0.386	(1.24, 6.94), 0.014*	(0.88, 5.15), 0.093
***Other Outcome*** *s*									
Smoking never permitted inside home	24 (23.5)	32 (33.0)	16 (17.2)	1.64	2.23	1.36	1.63	2.35	1.44
				(0.88, 3.06), 0.119	(1.14, 4.38), 0.020*	(0.68, 2.72), 0.388	(0.86, 3.10), 0.136	(1.17, 4.71), 0.016*	(0.71, 2.94), 0.312
You or others smoke at home	65 (63.7)	55 (56.7)	61 (65.6)	0.72	0.71	0.98	0.74	0.71	0.96
				(0.41, 1.28), 0.263	(0.40, 1.27), 0.247	(0.55, 1.76), 0.945	(0.42, 1.32), 0.312	(0.39, 1.28), 0.256	(0.53, 1.72), 0.878
I don’t want to maintain smoke-free home (SFH)	20 (19.6)	18 (18.6)	16 (17.2)	0.78	0.89	1.13	0.79	0.91	1.14
				(0.46, 1.33), 0.367	(0.51, 1.54), 0.666	(0.66, 1.93), 0.647	(0.48, 1.36), 0.371	(0.52, 1.56), 0.672	(0.68, 1.94), 0.653
SHS causes heart disease in adults	84 (82.4)	84 (86.6)	78 (83.9)	1.35	1.23	0.91	1.35	1.2	0.89
				(0.62, 2.93), 0.445	(0.55, 2.74), 0.618	(0.43, 1.92), 0.799	(0.62, 2.93), 0.455	(0.54, 2.70), 0.656	(0.42, 1.90), 0.770
SHS causes lung illnesses in children	97 (95.1)	94 (96.9)	85 (91.4)	1.58	2.91	1.84	1.55	2.83	1.83
				(0.37, 6.81), 0.537	(0.75, 11.34), 0.123	(0.58, 5.84), 0.300	(0.36, 6.70), 0.559	(0.72, 11.09), 0.135	(0.57, 5.87), 0.309
Smoking causes stroke	74 (72.6)	73 (75.3)	69 (74.2)	1.12	1.11	1.23	1.12	1.04	0.93
				(0.60, 2.11), 0.724	(0.58, 2.11), 0.750	(0.65, 2.31), 0.522	(0.59, 2.12), 0.722	(0.54, 2.01), 0.905	(0.49, 1.76), 0.818
Smoking causes heart attack	79 (77.5)	82 (84.5)	71 (76.3)	1.59	1.69	1.06	1.56	1.68	1.07
				(0.77, 3.27), 0.206	(0.82, 3.51), 0.157	(0.55, 2.07), 0.855	(0.76, 3.23), 0.228	(0.81, 3.51), 0.167	(0.55, 2.11), 0.834

*p < 0.05.

^Controlling age, ethnicity, education level and occupation.

**Table 5:** Intervention effects on household SHS exposure, smoke-free homes enforcement, and other related outcomes among mothers of newborns at 6 months

	N (%)			Crude OR (95% CI), p			Adjusted OR^ (95% CI), p		
	I-A (n = 104)	I-B (n = 100)	Control (n = 97)	I-B vs I-A	I-B vs Control	I-A vs Control	I-B vs I-A	I-B vs Control	I-A vs Control
***Primary Outcomes***									
Exposure to household SHS	70 (67.3)	61 (61.0)	64 (66.0)	0.76	0.81	1.06	0.78	0.79	1.02
				(0.43, 1.35), 0.348	(0.45, 1.44), 0.468	(0.59, 1.91), 0.842	(0.43, 1.4), 0.405	(0.44, 1.43), 0.444	(0.56, 1.85), 0.95
***Other Outcomes***									
Smoking never permitted inside home	22 (21.2)	24 (24.0)	18 (18.6)	1.18	1.39	1.18	1.12	1.40	1.25
				(0.61, 2.27), 0.627	(0.70, 2.76), 0.352	(0.59, 2.36), 0.645	(0.57, 2.18), 0.747	(0.70, 2.80), 0.343	(0.62, 2.54), 0.53
I don’t want to maintain smoke-free home (SFH)	5 (4.8)	4 (4.0)	2 (2.1)	0.82	1.98	2.40	0.89	2.05	2.31
				(0.22, 3.16), 0.779	(0.35, 11.06), 0.437	(0.45, 12.66), 0.303	(0.23, 3.45), 0.861	(0.36, 11.49), 0.417	(0.43, 12.33), 0.327
SHS causes heart disease in adults	90 (86.5)	91 (91.0)	78 (80.4)	1.57	2.46	1.57	1.49	2.45	1.64
				(0.65, 3.82), 0.317	(1.05, 5.76), 0.037*	(0.74, 3.33), 0.244	(0.61, 3.66), 0.383	(1.04, 5.76), 0.040*	(0.77, 3.53), 0.203
SHS causes lung illnesses in children	103 (99.0)	100 (100)	95 (98.0)	N/A	N/A	2.15	N/A	N/A	3.42
				N/A	N/A	(0.19, 24.09), 0.535	N/A	N/A	(0.22, 53.23), 0.380
Smoking causes stroke	74 (71.2)	75 (75.0)	68 (70.1)	1.22	1.28	1.05	1.13	1.27	1.12
				(0.65, 2.26), 0.536	(0.68, 2.4), 0.442	(0.57, 1.93), 0.87	(0.60, 2.12), 0.706	(0.67, 2.38), 0.465	(0.61, 2.07), 0.715
Smoking causes heart attack	86 (82.7)	85 (85.0)	75 (77.3)	1.19	1.66	1.40	1.06	1.67	1.58
				(0.56, 2.51), 0.655	(0.80, 3.44), 0.17	(0.70, 2.81), 0.341	(0.49, 2.27), 0.885	(0.80, 3.50), 171	(0.78, 3.23), 0.207

*p < 0.05.

^Control age, ethnicity, education level and occupation.

**Table 6:** Intervention effects on household SHS exposure, smoke-free homes enforcement, and other related outcomes among mothers of newborns at 12 months.

	N (%)			Crude OR (95% CI), p			Adjusted OR^ (95% CI), p		
	I-A (n = 104)	I-B (n = 97)	Control (n = 96)	I-B vs I-A	I-B vs Control	I-A vs Control	I-B vs I-A	I-B vs Control	I-A vs Control
***Primary Outcomes***									
Exposure to household SHS	70 (67.3)	59 (60.8)	72 (75.0)	0.75	0.52	0.69	0.77	0.53	0.69
				(0.42, 1.34), 0.339	(0.28, 0.96), 0.036*	(0.37, 1.27), 0.232	(0.43, 1.39), 0.392	(0.29, 0.99), 0.046*	(0.37, 1.28), 0.237
***Other Outcomes***									
Smoking never permitted inside home	28 (26.9)	31 (32.0)	25 (26.3)	1.13	1.22	1.08	1.13	1.25	1.11
				(0.53, 2.38), 0.754	(0.56, 2.63), 0.614	(0.50, 2.35), 0.842	(0.52, 2.46), 0.754	(0.56, 2.79), 0.582	(0.5, 2.47), 0.805
I don’t want to maintain smoke-free home (SFH)	7 (6.7)	13 (13.4)	7 (7.3)	2.14	1.97	0.92	2.01	2.03	1.01
				(0.82, 5.62), 0.121	(0.75, 5.17), 0.170	(0.31, 2.72), 0.877	(0.75, 5.43), 0.166	(0.75, 5.45), 0.162	(0.33, 3.05), 0.992
SHS causes heart disease in adults	89 (85.6)	83 (85.6)	75 (78.1)	1.00	1.66	1.66	0.94	1.69	1.79
				(0.45, 2.20), 0.998	(0.79, 3.50), 0.182	(0.80, 3.45), 0.173	(0.43, 2.09), 0.887	(0.8, 3.59), 0.171	(0.85, 3.76), 0.123
SHS causes lung illnesses in children	100 (96.2)	94 (96.9)	87 (90.6)	1.28	3.28	2.56	1.07	3.36	3.14
				(0.28, 5.87), 0.752	(0.86, 12.51), 0.082	(0.76, 8.62), 0.128	(0.23, 5.01), 0.930	(0.86, 13.10), 0.081	(0.91, 10.88), 0.071
Smoking causes stroke	82 (78.9)	72 (74.2)	73 (76.0)	0.77	0.91	1.17	0.77	0.94	1.21
				(0.40, 1.49), 0.440	(0.47, 1.74), 0.771	(0.60, 2.28), 0.635	(0.40, 1.51), 0.452	(0.49, 1.82), 0.857	(0.62, 2.38), 0.571
Smoking causes heart attack	89 (85.6)	83 (85.6)	79 (82.3)	1.00	1.28	1.28	0.96	1.37	1.43
				(0.45, 2.20), 0.998	(0.59, 2.76), 0.536	(0.60, 2.72), 0.527	(0.43, 2.15), 0.911	(0.62, 3.02), 0.435	(0.66, 3.12), 0.365

*p < 0.05.

^Control age, ethnicity, education level and occupation.

Regarding other outcomes, participants in I-B group are more likely (adjusted OR: 2.35, 95% CI: 1.17–4.71; p = 0.016) to report “smoking never permitted inside home” compared to participants in control group at 12 months (Table 4). In addition, participant mothers in I-B group are more likely (adjusted OR: 2.45, 95% CI: 1.04–5.76; p = 0.040) to be aware that “secondhand smoking causes heart disease in adults” compared to participants in control group at 6 months (Table 5).

## Discussion

Our study was one of the first known experimental research studies to examine the effects of a mobile phone text message-based intervention on the smoking behaviors of fathers and self-reported SHS exposure of mothers of newborns during the early postnatal period. Compared to the control group, the group that received health education counseling combined with a text message service had almost tripled the rate of self-reported smoking abstinence at the 6-month follow-up visit (risk difference: 12.7%); the risk difference remained high (13.0%) at the 12-month follow-up visit. Meanwhile, the self-reported SHS exposure among non-smoking mothers whose husbands received text message interventions was significantly reduced at the 12-month follow-up. These findings suggest that the addition of an mHealth element to interventions with in-person counseling and provision of educational materials effectively aided in creating smoke-free homes and promoted smoking cessation among fathers of newborns.

The differential increase in smoking cessation rates among the mHealth intervention group in our current study confirms the findings of earlier research on smoking cessation interventions using text-based mobile phone services^26^, including two recent RCTs conducted in China. One RCT included a large smoker population in China and found a significant increase in the self-reported past-7-day abstinence rate among the group that received a high-frequency of text contacts compared to the group that received low-frequency text contact, although the magnitude of the difference was relatively small^30^. The other RCT targeted a subpopulation of Chinese smokers, adolescent smokers aged 16 to 19 years, and found that the text messaging intervention group had a significantly higher rate of smoking reduction compared to the control group^31^. Our study adds novel insight to the current research on mHealth smoking cessation interventions in China because it targeted a unique subgroup of Chinese smokers (i.e. fathers of newborn babies) and supported current evidence that mHealth is a promising intervention for future practice. In China, more than 90% of population now have mobile phones^32^, allowing for a more accessible and efficient means of delivering health information. Based on this study and the findings from other mHealth interventions, text-messaging interventions should be promoted more aggressively as a tool to protect women and children from second-hand smoking exposure. In addition, based on past and present evolution of mobile phone-based applications, it may be useful to consider other mobile platforms (e.g. WeChat, a commonly used platform in China) for future mHealth interventions^33^.

Our study implies the important role of non-smoking women in helping their spouses quit smoking by focusing the major concerns with smoking on the health of their children. Unlike a number of previous smoking cessation studies in China targeted only to male smokers, our study also targeted non-smoking mothers and encouraged them to be change agents. Non-smoking mothers of the newborns and their smoking husbands received the counseling at the same time. This arrangement promoted spousal interaction and support, which has been found to be effective in helping smokers quit or reduce smoking^34–36^. Although complete cessation is not the norm for Chinese smokers, when the health of unborn or newborn babies is at risk, smoking fathers usually become more susceptible to opinions from their spouses who are primarily responsible for caring for their children. For instance, a RCT study conducted in Guangzhou, China found that simple health advice provided by obstetricians to non-smoking pregnant women was effective in helping their partner reduce consumption or quit smoking completely^37^. A similar study design was applied to parents of sick children in Hong Kong and found that health education provided by nurses to mothers of sick children triggered quit attempts by the husband in the short term and aided with increased cessation^38^. Thus, women should be mobilized in interventions designed to protect children, especially at the most vulnerable stages of child growth and development.

Our study faces limitations and the results should be interpreted with caution. The first limitation is the reliance on self-reported data in our approach to evaluate outcomes. Such self-report may be biased and lead to inaccurate measures of smoking abstinence and nicotine exposure. Biochemical verification with cotinine or carbon monoxide tests is desirable for future studies, even though bio-samples, such as hair, blood, saliva, and urine, can be difficult to obtain^39^. Second, our study is based on a relatively small sample, which may have limited the ability to detect a true effect even when it existed. As the statistical power analysis suggested, the sample size of this study only allow us to detect association with odds ratio larger than 2.0 or smaller than 0.5 at 80% statistical power and 5% significance, which may explain why we failed to detect any significant difference in the outcomes neither between control group and traditional intervention group, nor the difference between traditional intervention group and mHealth intervention group. Future research with a larger sample is merited. Third, the participants were recruited from two urban districts of Changchun, where residents were considered to maintain higher social economic status, education level and health status. Therefore, the sample may not be representative of the whole city. Finally, the content of the text messages sent to the participants in our trial lacked diversity. The vast majority of the messages were related to the health effects of smoking and SHS exposure and the health benefits of quitting. Cessation advice, such as making a public declaration, asking for social support, tips to cope with cravings, and using distraction techniques; and personalized messages that address individual needs were not included in our trial. A recent trial conducted in China found that text messages including cessation advice were more effective than messages that addressed health effects alone^30^. Future mHealth interventions should consider sending participants an increased variety of smoking cessation messages.

In summary, mHealth interventions with a text message component are an effective way to increase male smoking cessation and reduce mother and newborn SHS exposure in the home in China. Including female spouses as a supportive aid in these interventions can increase the success of cessation for husbands. Future studies should include measured nicotine consumption and exposure, other potential messaging platforms, an increased variety of smoking cessation messages, and cost-effective analysis of the mHealth intervention.

## References

[1] DiFranza JR, Aligne CA, Weitzman M (2004). Prenatal and postnatal environmental tobacco smoke exposure and children’s health. Pediatrics.

[2] Hofhuis W, De Jongste J, Merkus P (2003). Adverse health effects of prenatal and postnatal tobacco smoke exposure on children. Archives of disease in childhood.

[3] (US), O. o. S. a. H. The Health Consequences of Involuntary Exposure to Tobacco Smoke: A Report of the Surgeon General. (Centers for Disease Control and Prevention 2006). 20669524

[4] Anderson HR, Cook DG (1997). Passive smoking and sudden infant death syndrome: review of the epidemiological evidence. Thorax.

[5] California Environmental Protection Agency: Air Resources, B. (2005).

[6] Gehrman CA, Hovell MF (2003). Protecting children from environmental tobacco smoke (ETS) exposure: a critical review. Nicotine Tob Res.

[7] McMartin KI (2002). Lung tissue concentrations of nicotine in sudden infant death syndrome (SIDS). J Pediatr.

[8] Öberg M, Jaakkola MS, Woodward A, Peruga A, Prüss-Ustün A (2011). Worldwide burden of disease from exposure to second-hand smoke: a retrospective analysis of data from 192 countries. The Lancet.

[9] Li Q, Hsia J, Yang G (2011). Prevalence of smoking in China in 2010. New England Journal of Medicine.

[10] Gonhuan Y (2010). China wrestles with tobacco control. Bull World Health Organ.

[11] Zhang, L. *et al*. Peer Reviewed: Exposure to Secondhand Tobacco Smoke and Interventions Among Pregnant Women in China: A Systematic Review. *Preventing chronic disease* **12** (2015). 10.5888/pcd12.140377 PMC4372160 25789496

[12] Fu C, Chen Y, Wang T, Edwards N, Xu B (2008). Exposure to environmental tobacco smoke in Chinese new mothers decreased during pregnancy. Journal of clinical epidemiology.

[13] Kim SS (2012). A systematic review of smoking cessation intervention studies in China. Nicotine & Tobacco Research.

[14] Organization, W. H. Global Adult Tobacco Survey (GATS). Fact Sheet China: 2010. *Manila*: *World Health Organization* (2010).

[15] Goodman, J. *Tobacco in history and culture: an encyclopedia*. (Granite Hill Publishers, 2005).

[16] Mao A, Bristow K, Robinson J (2013). Caught in a dilemma: why do non-smoking women in China support the smoking behaviors of men in their families?. Health Educ Res.

[17] Wei, X. *et al*. Household smoking restrictions related to secondhand smoke exposure in Guangdong, China: A population representative survey. *nicotine & tobacco research*, ntt162 (2013). 10.1093/ntr/ntt162 24130143

[18] Abdullah AS (2015). Secondhand smoke exposure reduction intervention in Chinese households of young children: a randomized controlled trial. Academic pediatrics.

[19] Baheiraei A (2011). Reduction of secondhand smoke exposure among healthy infants in Iran: randomized controlled trial. Nicotine & tobacco research.

[20] Rosen LJ, Myers V, Winickoff JP, Kott J (2015). Effectiveness of Interventions to Reduce Tobacco Smoke Pollution in Homes: A Systematic Review and Meta-Analysis. International journal of environmental research and public health.

[21] Rosen LJ, Noach MB, Winickoff JP, Hovell MF (2012). Parental smoking cessation to protect young children: a systematic review and meta-analysis. Pediatrics.

[22] Schroer-Gunther M, Zhou M, Gerber A, Passon A (2011). Primary tobacco prevention in China-a systematic review. Asian Pac J Cancer Prev.

[23] Free C (2013). The effectiveness of mobile-health technology-based health behaviour change or disease management interventions for health care consumers: a systematic review. PLoS med.

[24] Irvine L (2012). Can text messages reach the parts other process measures cannot reach: an evaluation of a behavior change intervention delivered by mobile phone?. PLoS One.

[25] Kumar S (2013). Mobile health technology evaluation: the mHealth evidence workshop. American journal of preventive medicine.

[26] Spohr SA (2015). Efficacy of SMS Text Message Interventions for Smoking Cessation: A Meta-Analysis. J Subst Abuse Treat.

[27] Whittaker, R. *et al*. Mobile phone‐based interventions for smoking cessation. *The Cochrane Library* (2012). 10.1002/14651858.CD006611.pub3 23152238

[28] Mussener U (2016). Effectiveness of Short Message Service Text-Based Smoking Cessation Intervention Among University Students: A Randomized Clinical Trial. JAMA Intern Med.

[29] Redmon P, Koplan J, Eriksen M, Li S, Kean W (2014). The role of cities in reducing smoking in China. International journal of environmental research and public health.

[30] Augustson, E. *et al*. Text to Quit China: An mHealth Smoking Cessation Trial. *Am J Health Promot*; DOI:10.4278/ajhp.140812-QUAN-399 (2016). 10.4278/ajhp.140812-QUAN-399 PMC4935631 26730560

[31] Shi HJ, Jiang XX, Yu CY, Zhang Y (2013). Use of mobile phone text messaging to deliver an individualized smoking behaviour intervention in Chinese adolescents. J Telemed Telecare.

[32] World Bank. Mobile cellular subscriptions (per 100 people). http://data.worldbank.org/indicator/IT.CEL.SETS.P2?locations=CN (2016).

[33] Lien CH, Cao Y (2014). Examining WeChat users’ motivations, trust, attitudes, and positive word-of-mouth: Evidence from China. Computers in Human Behavior.

[34] Hemsing N, Greaves L, O’Leary R, Chan K, Okoli C (2012). Partner support for smoking cessation during pregnancy: A systematic review. Nicotine & Tobacco Research.

[35] Kegler MC, Escoffery C, Groff A, Butler S, Foreman A (2007). A qualitative study of how families decide to adopt household smoking restrictions. Family & community health.

[36] Kegler, M. C. *et al*. Pilot study results from a brief intervention to create smoke-free homes. *Journal of environmental and public health* **2012** (2012). 10.1155/2012/951426 PMC3362929 22675374

[37] Loke AY (2000). Exposure to and actions against passive smoking in non‐smoking pregnant women in Guangzhou, China. Acta obstetricia et gynecologica Scandinavica.

[38] Chan SS, Leung GM, Wong DC, Lam T-H (2008). Helping Chinese fathers quit smoking through educating their nonsmoking spouses: a randomized controlled trial. American Journal of Health Promotion.

[39] Florescu A (2009). Methods for quantification of exposure to cigarette smoking and environmental tobacco smoke: focus on developmental toxicology. Therapeutic drug monitoring.

